# Intrinsic motivation in patients with Parkinson’s disease: a neuropsychological investigation of curiosity using dopamine transporter imaging

**DOI:** 10.1007/s10072-020-04968-4

**Published:** 2021-01-07

**Authors:** Yayoi Shigemune, Iori Kawasaki, Akira Midorikawa, Toru Baba, Atsushi Takeda, Nobuhito Abe

**Affiliations:** 1 Research and Development Initiative, Chuo University, 742-1 Higashinakano, Hachioji-shi, Tokyo, 192-0393 Japan; 2 Institute of Cultural Science, Chuo University, Tokyo, Japan; 3 Department of Rehabilitation, National Hospital Organization Sendai-Nishitaga Hospital, Sendai, Japan; 4 Department of Psychology, Faculty of Letters, Chuo University, Tokyo, Japan; 5 Department of Neurology, National Hospital Organization Sendai-Nishitaga Hospital, Sendai, Japan; 6 Kokoro Research Center, Kyoto University, Kyoto, Japan

**Keywords:** Parkinson’s disease, Intrinsic motivation, Curiosity, Striatum, Dopamine transporter

## Abstract

Both intrinsic and extrinsic motivation are believed to involve brain regions that are innervated by the dopaminergic pathway. Although dopaminergic neurons in the midbrain deteriorate in Parkinson’s disease (PD), it remains unclear whether intrinsic motivation is impaired in PD patients. To address this issue, we investigated intrinsic motivation in PD patients using a task designed to assess the “Pandora effect,” which constitutes a curiosity for resolving uncertainty, even if this curiosity is likely to result in negative consequences. Twenty-seven PD patients and 27 age-matched healthy controls (HCs) completed a curiosity task in which they were required to decide either to view or skip negative pictures (e.g., snakes, spiders) and an examination battery that included the Mini-Mental State Examination, a verbal fluency test, the Trail Making Test, 10-word recall tests, and questionnaires for behavioral inhibition/activation and depression. DaTSCAN images to assess the distribution of dopamine transporters in the striatum were acquired only from PD patients. The results revealed that PD patients, relative to the HCs, viewed the pictures less frequently under both the certain and uncertain conditions. However, both the PD patients and HCs viewed the pictures at a higher frequency under the uncertain condition than under the certain condition. In the PD patients, the proportion of pictures viewed under the certain condition was positively correlated with the distribution of dopamine transporters in the striatum. These results suggest that despite the overall decreasing level of interest in viewing negative pictures, the motivation to resolve uncertainty is relatively intact in PD patients.

## Introduction

The major neuropathological feature of Parkinson’s disease (PD) is the degeneration of dopaminergic neurons in the substantia nigra and ventral tegmental area of the midbrain [1, 2]. The dopaminergic neurons in the substantia nigra and ventral tegmental area project to the striatum, and these dopaminergic projections are included in the reward circuit [3]. Accordingly, striatal dopaminergic transmissions deteriorate in PD patients [4], and these localized impairments in the dopaminergic reward pathway induce abnormal reward-related activation and processing among PD patients [5].

Motivation is a key process that reflects the functions of reward-related brain regions. Motivation can be defined as a process that determines the direction (i.e., defining which goals an organism seeks to approach or avoid) and energization (i.e., the mobilization of resources to carry out an action) of behavior [6, 7] and can be classified into extrinsic and intrinsic motivation. While the former shapes goal-directed behavior to obtain external rewards, such as food and drink, monetary incentives, and social approval, the latter occurs without external rewards because the action itself is inherently interesting, enjoyable, and satisfying [8]. Curiosity is a form of intrinsic motivation that is key in fostering spontaneous exploration [9, 10]. Previous neuroimaging studies suggested that intrinsic motivation, including curiosity, and extrinsic motivation are involved in the dopaminergic reward pathway [11–14].

Despite accumulated evidence indicating that intrinsic and extrinsic motivation share a common neural basis, it remains unclear whether intrinsic motivation deteriorates in PD patients accompanied by the loss of striatal dopaminergic transmission. We investigated this unexplained issue using ^123^I-ioflupane single-photon emission computed tomography (SPECT) (DaTSCAN) images to assess the distribution of dopamine transporters in the striatum, combined with a task designed to assess the “Pandora effect,” which constitutes a curiosity for resolving uncertainty, even if this curiosity is likely to result in negative consequences [15]. In this task, participants had to decide whether to view or skip negative pictures under a condition in which the names of objects in the pictures were known (i.e., the certain condition) or in which just a question mark was presented and the contents of the pictures were uncertain (i.e., the uncertain condition). An earlier study indicated that participants were motivated to view even negative pictures, and more chose to view the negative pictures when the contents of the pictures were uncertain [15]. This task therefore enabled us to investigate curiosity characterized by an overall interest in viewing negative pictures and to further determine whether the motivation to resolve uncertainty affects motivated behavior. We hypothesized that PD patients, relative to healthy controls (HCs), would view negative pictures at a reduced rate and exhibit no effect of stimulus uncertainty owing to a decrease in the distribution of dopamine transporters in the striatum.

## Materials and methods

### Participants

Twenty-seven PD patients without dementia (mean age: 69.5 years; SD: 6.1 years; 7 men and 20 women) who were hospitalized in Sendai-Nishitaga National Hospital participated in this study. The inclusion criteria for patients in this study were as follows: age between 50 and 80 years, age at onset above 40 years, Hoehn-Yahr stage from 1 to 4, and a score of 24 or higher on the Mini-Mental State Examination (MMSE) [16]. The exclusion criteria were as follows: a medical history of disease of the central nervous system not directly related to PD (e.g., stroke, head injury, epilepsy), a history of deep brain stimulation surgery, a history of familial PD, concurrent psychiatric illness, such as schizophrenia or manic depression, a documented or suspected history of drug abuse and/or alcoholism, diabetes mellitus, and major abnormalities on brain MRI scans, such as cerebral infarction or tumor. No patient had self-control problems associated with dopamine dysregulation syndrome, such as addiction to gambling. The motor symptoms of the PD patients were evaluated using Hoehn-Yahr staging [17] and the Unified Parkinson’s Disease Rating Scale (UPDRS) part III [18]. Scores for the UPDRS part III were recorded while the patients were on medication. Twenty-nine HCs (mean age: 69.9 years; SD: 3.8 years; 10 men and 19 women) were recruited from a job placement center for elderly people with payment. The HCs had no medical history of psychiatric or neurological disease. Data from 27 HCs (mean age: 70.0 years; SD: 3.9 years; 10 men and 17 women) were used in subsequent data analyses because one participant refused to participate in the experimental task, which included emotionally negative pictures, and another participant was extremely disgusted by a snake shown in the experimental task. The optimal sample size (i.e., 27 for each group) was determined based on a G*Power analysis [19] using a power of 0.95, a medium effect size of *f* = 0.25 for analysis of variance (ANOVA) to test within-between interactions, and an *α* level of 0.05; data collection ceased when this number was satisfied for the analysis. The demographic data of the PD patients and HCs are summarized in Table 1. No significant differences were observed between the PD patients and HCs with respect to age (*t*(44.3) = − 0.32, *p* = 0.75, *r* = 0.05), years of education (*t*(52) = 0.06, *p* = 0.95, *r* = 0.01), or sex (*χ*^*2*^ = 0.77, *p* = 0.38, *V* = 0.12). The protocol was approved by the ethics committees of Sendai-Nishitaga National Hospital and Chuo University. All subjects gave written informed consent in accordance with the Declaration of Helsinki.

**Table 1:** Demographic data

	PD patients (*n* = 27)	HCs (*n* = 27)	
Age (mean ± SD)	69.5 ± 6.1	70.0 ± 3.9	† n.s.
Sex (male:female)	7:20	10:17	‡ n.s.
Years of education (mean ± SD)	13.8 ± 2.5	13.8 ± 1.9	† n.s.
Years of PD (mean ± SD)	9.1 ± 6.3	-	
Levodopa equivalent dose (mg/day) (mean ± SD)	661.1 ± 183.8	-	
UPDRS part III (mean ± SD)	22.6 ± 13.9	-	
Hoehn-Yahr stage (mean ± SD)	2.6 ± 0.6	-	
Stage I (*n*)	0	-	
Stage II (*n*)	13	-	
Stage III (*n*)	13	-	
Stage IV (*n*)	1	-	

*PD* Parkinson’s disease, *HCs* healthy controls, *SD* standard deviation, *UPDRS* Unified Parkinson’s Disease Rating Scale, *n.s.* not significant

†*t* test

‡Chi-square test

### Examination battery

An examination battery that included the MMSE [16], phonemic and semantic verbal fluency (VF) tests, the Trail Making Test (TMT) [20], the 10-word recall item of the Alzheimer’s Disease Assessment Scale (ADAS) [21], behavioral inhibition system/behavioral activation system (BIS/BAS) scales [22], and the Hospital Anxiety and Depression Scale (HADS) [23] was administered to the participants.

### Experimental curiosity task

**Fig. 1:**
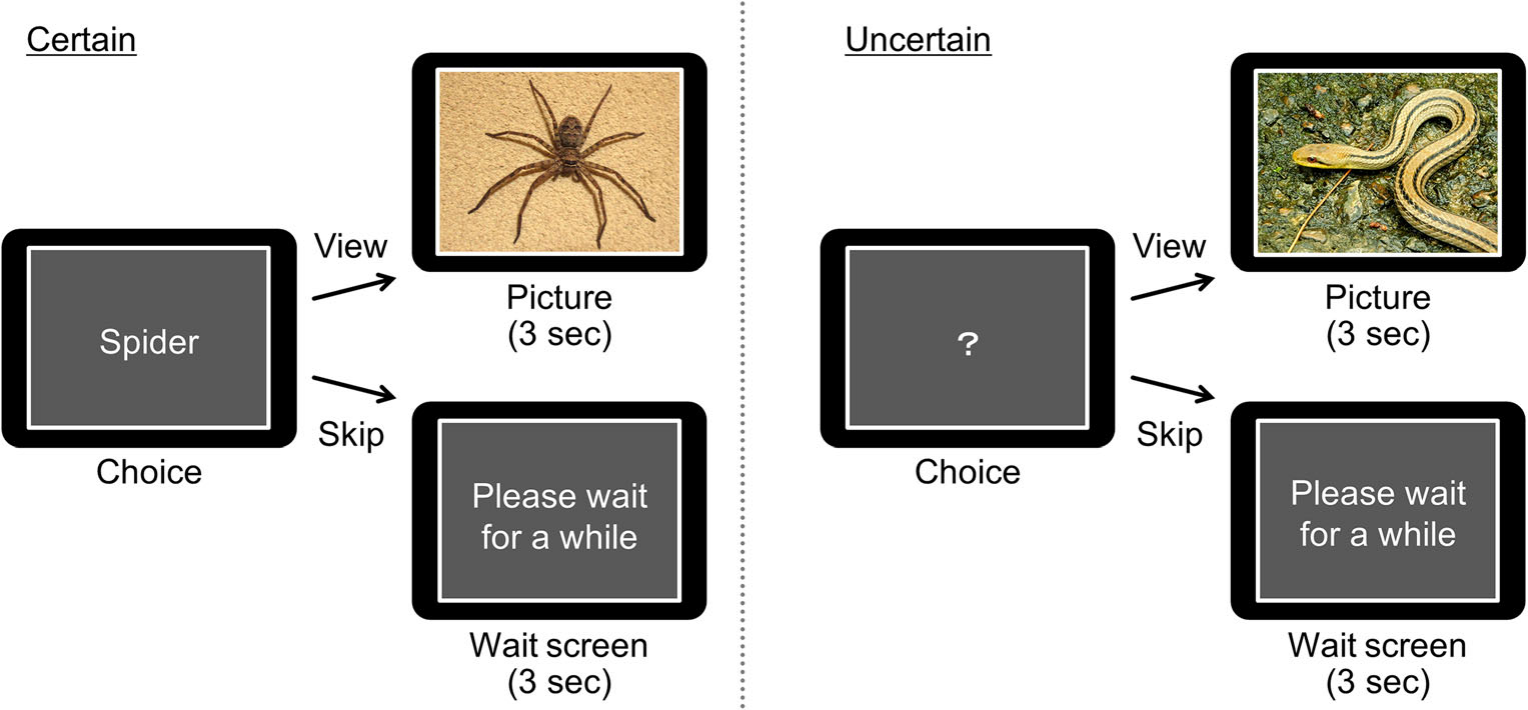
Experimental task design. Under the certain condition, the name of the object in a picture was presented on a rectangle covering the picture, whereas a question mark was presented on the rectangle covering the picture under the uncertain condition. Participants were required to decide either to view or skip the picture. If the participants chose to view the picture, the picture was presented for 3 s. If the participants chose to skip the picture, the message “please wait for a while” was presented for 3 s. Note that these pictures are not the actual stimuli used as we do not have the right to include pictures from IAPS in this publication

We modified the task used in study 4 of the report by Hsee and Ruan [15]. Eight negative pictures of a snake, a spider, a dog, a cockroach, a bear, a shark, a rat, and flies were selected from the International Affective Picture System for the experimental task (mean valence score: 3.79, SD: 0.32; mean arousal score: 5.78, SD: 0.80) [24]. These pictures were divided into two homogeneous sets in valence (*t*(6) = − 0.41, *p* = 0.70, *r* = 0.17) and arousal (*t*(6) = 0.02, *p* = 0.98, *r* = 0.01). The image sets and experimental conditions were combined in a counterbalanced manner. In each trial of the experimental task (Fig. 1), a rectangle covering the negative picture was presented on a computer screen. On the rectangle, the name of the hidden animal was indicated under the certain condition, whereas a question mark was presented under the uncertain condition. The participants had to decide whether to view or skip the picture. If participants chose to view the picture, the rectangle was removed, and the negative picture was presented for 3 s. If participants chose to skip the picture, a message advising “please wait for a while” was presented in the rectangle for 3 s. Each of the eight pictures was randomly presented 5 times in the experimental task. Before the experimental task, the participants performed a short practice session in which they were presented with two stimuli from the certain condition and two stimuli from the uncertain condition. They were required to choose to view the pictures in all of these practice trials to learn the relationship between the names or question mark and the hidden animals.

### DaTSCAN image acquisition

DaTSCAN images (GE Healthcare, USA) were acquired using a SPECT/CT scanner (Symbia Intevo 6, Siemens, Germany) only for the PD patients. Imaging was performed for 30 min at approximately 180 min after intravenous injection of ^123^I-ioflupane (167 MBq) with a matrix size of 128 × 128, pixel size of 3.3 mm, slice thickness of 3 mm, and energy window of 159 keV ± 15%. SPECT data were reconstructed with an iterative algorithm using 3D-ordered subset expectation maximization with 10 iterations and six subsets. Non-contrast CT (150 mAs with AEC/130 kV) was also performed using the same SPECT/CT machine for attenuation correction, scatter correction, and anatomical coregistration. CT images were reconstructed in the axial plane, with a thickness of 5 mm and in a 512 × 512 matrix (field of view = 300 mm × 300 mm).

The distribution of dopamine transporters in the right and left striata was analyzed using the Scenium software (Siemens, Germany). Each subject’s DaTSCAN image was coregistered with their CT image and normalized to the Montreal Neurological Institute space via affine registration using the CT template provided by the Scenium software. The automated region of interest (ROI) definition in Scenium enables the calculation of the distribution volume ratio of dopamine transporters in the right and left striata normalized to the whole brain [25]. Scaled striatal uptake images were obtained by dividing each voxel value by the 75th percentile voxel value within the reference brain. The definitions of the ROIs were validated through visual inspection by medical radiology technicians unrelated to this study.

### Data analyses

To confirm whether curiosity was impaired in the PD patients, the proportions of pictures viewed were analyzed using two-way ANOVA with group (PD patients vs. HCs) and condition (certain vs. uncertain) as factors. Two-sample *t* tests of the scores of the examination battery, including those of the MMSE, TMT, phonemic VF test, semantic VF test, 10-word recall test of the ADAS (averaged over three attempts), BIS/BAS, and HADS, were conducted to compare the general cognitive function, frontal lobe function, episodic memory, personality traits, and mental states between the PD patients and HCs. To further confirm whether the differences identified by the *t* tests (i.e., TMT, BAS, anxiety, and depression) affected curiosity, we examined whether these measures worked as potential mediators between a factors of group (PD patients vs. HCs) as an independent variable and the proportion of pictures viewed as a dependent variable. Using the PROCESS SPSS macro [26], we employed a bootstrapping method with bias-corrected confidence intervals to test the significance of potential mediation effects [27, 28]. Rather than providing formal *p* values, this method uses bootstrap confidence intervals of an indirect effect to test for significance, whereby confidence intervals that do not contain 0 indicate a mediation effect. Bootstrap analyses and estimates were based on 10,000 bootstrap samples. IBM SPSS Statistics version 22 was used for the analyses (IBM, USA). To investigate the relationship between curiosity and dopamine neurotransmission in the striatum, correlation analysis between the proportion of pictures viewed and the distribution volume ratio in the right and left striata were performed for the PD patients. We used a one-tailed test for the correlation analysis because our hypothesis regarding the association between performance in the experimental curiosity task and the dopamine transporter is unidirectional (i.e., lower curiosity with lower distribution volume ratio of dopamine transporter). The DaTSCAN image of one PD patient was missing because they had undergone DaTSCAN imaging at another hospital immediately before their hospitalization. Thus, the correlation analysis used neuroimaging data from 26 rather than 27 patients.

## Results

### Scores of the examination battery

The results of the examination battery are shown in Table 2. Two-sample *t* tests showed no significant differences in the MMSE (*t*(52) = − 0.58, *p* = 0.56, *r* = 0.08), phonemic VF test (*t*(52) = − 1.86, *p* = 0.07, *r* = 0.25), semantic VF test (*t*(52) = − 1.55, *p* = 0.13, *r* = 0.21), 10-word recall test of the ADAS (*t*(52) = − 0.06, *p* = 0.95, *r* = 0.01), or BIS (*t*(52) = − 1.76, *p* = 0.08, *r* = 0.24) scores, but significant differences in TMT (PD patients > HCs) (*t*(38.7) = 3.17, *p* < 0.01, *r* = 0.40), BAS (PD patients < HCs) (*t*(52) = − 3.06, *p* < 0.01, *r* = 0.39), anxiety (PD patients > HCs) (*t*(52) = 2.39, *p* < 0.05, *r* = 0.31), and depression (PD patients > HCs) (*t*(52) = 2.41, *p* < 0.05, *r* = 0.32) scores were found.

**Table 2:** Examination battery results (mean ± SD)

	PD patients	HCs	
MMSE (out of 30)	28.1 ± 2.0	28.4 ± 1.8	† n.s.
VF			
Phonemic VF (n)	9.0 ± 3.0	10.5 ± 2.6	† n.s.
Semantic VF (n)	14.0 ± 4.1	16.3 ± 6.3	† n.s.
TMT			
Part A (sec)	64.7 ± 22.5	43.0 ± 11.5	† **
Part B (sec)	140.1 ± 71.9	78.4 ± 33.2	† **
Part B - part A (sec)	75.4 ± 58.3	35.5 ± 29.8	† **
Word recall (out of 10)	7.0 ± 1.4	7.0 ± 1.2	† n.s.
BIS/BAS scales			
BIS	15.4 ± 2.6	16.9 ± 3.4	† n.s.
BAS	32.7 ± 6.8	37.7 ± 5.1	† **
HADS			
Anxiety	6.9 ± 3.9	4.6 ± 3.3	† *
Depression	8.3 ± 3.3	6.2 ± 3.9	† *

*PD* Parkinson’s disease, *HCs* healthy controls, *SD* standard deviation, *MMSE* Mini-Mental State Examination, *VF* verbal fluency, *TMT* Trail Making Test, *ADAS* Alzheimer’s Disease Assessment Scale, *BIS* behavioral inhibition system, *BAS* behavioral activation system, *HADS* Hospital Anxiety and Depression Scale, *n.s*. not significant

**p < 0.01

*p < 0.05

†*t* test

### Experimental curiosity task

**Fig. 2:**
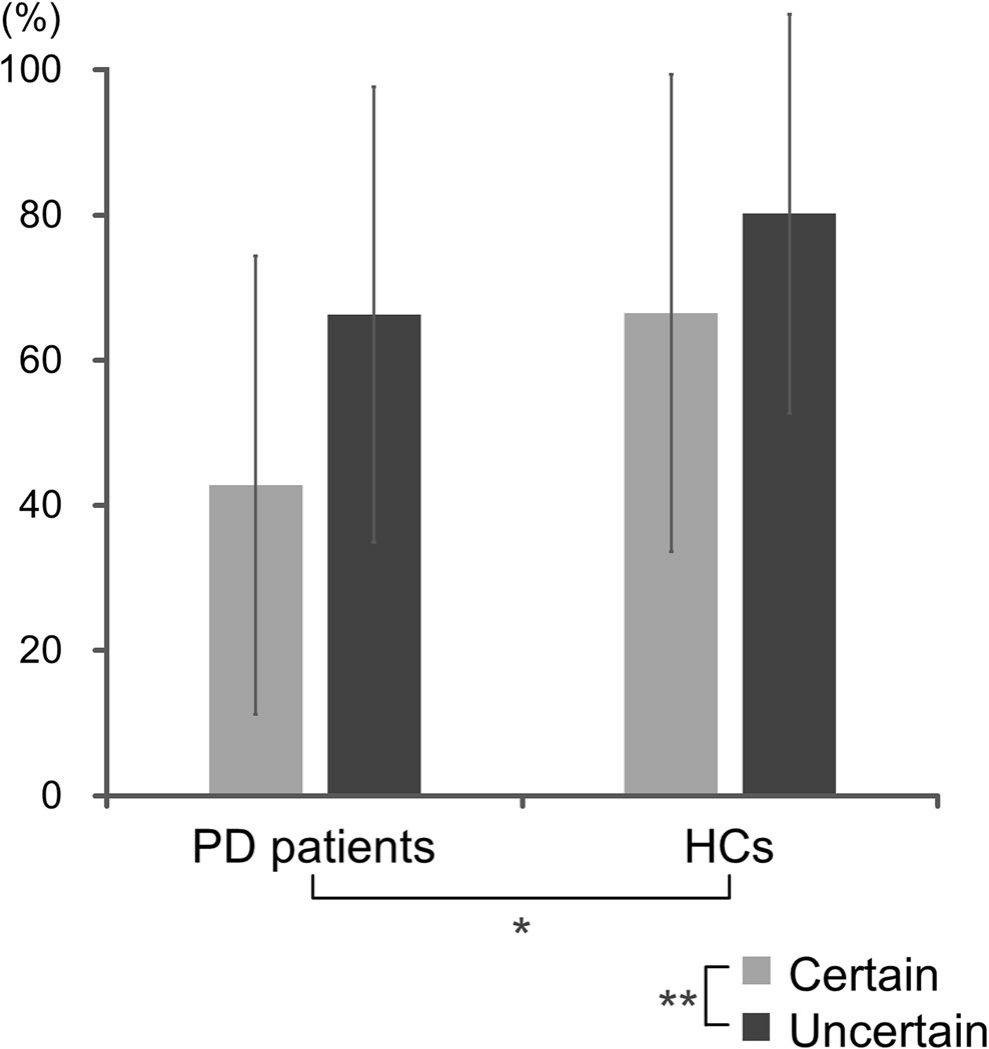
Proportions of pictures viewed in the experimental task. Error bars represent standard deviations. **p < 0.01, *p < 0.05

The mean proportions of pictures viewed under certain and uncertain conditions were 42.8% (SD: 31.8) and 66.3% (SD: 31.4) for PD patients and 66.5% (SD: 32.8) and 80.2% (SD: 27.5) for HCs, respectively. In a two-way ANOVA of the proportions of pictures viewed with group and condition as factors, the main effects of group (PD patients < HCs) (*F*(1,52) = 6.64, *p* < 0.05, *ηp*^2^ = 0.11) and condition (certain < uncertain) (*F*(1,52) = 20.0, *p* < 0.01, *ηp*^2^ = 0.28) were significant, but the interaction between group and condition was not significant (*F*(1,52) = 1.39, *p* = 0.24, *ηp*^2^ = 0.03) (Fig. 2).

### Mediation analysis of proportions of pictures viewed

TMT, BAS, anxiety, and depression scores differed significantly between the PD patients and HCs. These group differences might have contributed to the differences in the proportions of pictures viewed between PD patients and HCs. To address this issue, these scores were used as variables in a mediation analysis of the proportions of pictures viewed with a factor of group as an independent variable. In the absence of an interaction between group and condition, the overall proportion of pictures viewed (collapsed across the certain and uncertain conditions) was used as the dependent variable. In the mediation analysis, the bias-corrected 95% confidence interval for the indirect effect size included 0 for all factors (− 5.1 to 7.4 for TMT score, − 9.5 to 2.9 for BAS score, − 3.1 to 7.9 for anxiety score, and − 9.8 to 2.1 for depression score), indicating TMT, BAS, anxiety, and depression scores had a non-significant indirect effect on the group difference in proportion of pictures viewed.

### Correlation analysis of distribution volume ratios

**Fig. 3:**
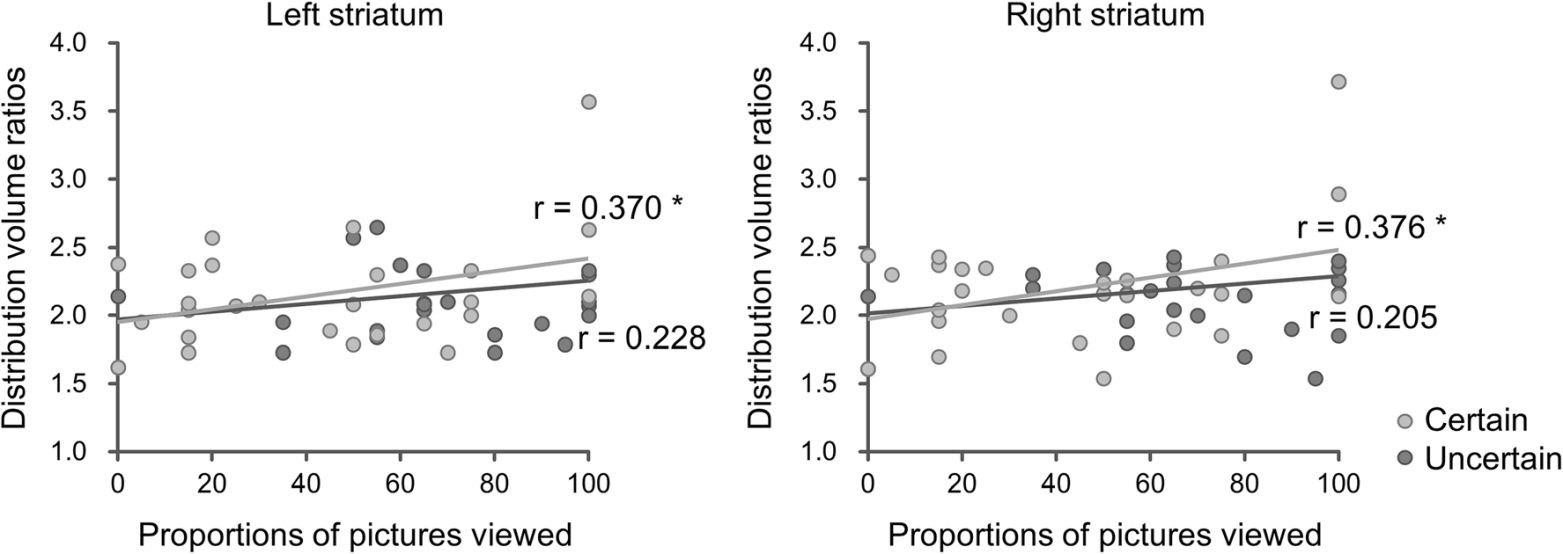
Correlations between distribution volume ratios in the right and left striata and proportion of pictures viewed under the certain and uncertain conditions. *p < 0.05, one-tailed

The proportions of pictures viewed were collapsed across the certain and uncertain conditions because the PD patients viewed fewer pictures than did the HCs, regardless of the condition. In the PD patients, analysis of the proportion of pictures viewed and the distribution volume ratios of the dopamine transporter in the right and left striata revealed significant correlations (right: *r* = 0.35, *p* = 0.04, one-tailed; left: *r* = 0.36, *p* = 0.04, one-tailed). Further analysis revealed that this significant correlation between the proportion of pictures viewed and the dopamine transporter was driven mainly by results from trials with the certain condition (right: *r* = 0.38, *p* = 0.03, one-tailed; left: *r* = 0.37, *p* = 0.03, one-tailed) rather than by those from trials with the uncertain condition (right: *r* = 0.21, *p* = 0.16, one-tailed; left: *r* = 0.23, *p* = 0.13, one-tailed) (Fig. 3).

## Discussion

In the present study, an experimental task examining the “Pandora effect” was administered to investigate whether curiosity declined in PD patients. The PD patients viewed fewer pictures than did the HCs regardless of whether the pictures’ contents were specified (i.e., certain condition) or not (i.e., uncertain condition), indicating that an overall interest in seeking information was diminished in the PD patients. We confirmed that these effects could not be ascribed to group differences in TMT, BAS, anxiety, and depression scores. We propose that the effects observed here are derived from decreased novelty seeking. Consistent with this interpretation, earlier studies of personality traits have suggested that novelty seeking is diminished in PD patients [29, 30].

The distribution volume ratios of dopamine transporters in the right and left striata were significantly correlated with the proportions of pictures viewed, indicating a close relationship between interest in seeking information and dopaminergic function. Earlier studies have also suggested a relationship between the dopaminergic system and novelty seeking [31–33]. For example, a pharmacological intervention study indicated that novelty seeking declined in never-medicated PD patients, whereas diminished novelty seeking was alleviated with dopaminergic medication [33]. We note that the present significant correlation was mainly driven by data from the certain condition but not from the uncertain condition. We speculate that decisions made under the certain condition were driven by an interest in viewing negative pictures, whereas decisions made under the uncertain condition were driven not only by overall interest but also by stimulus uncertainty, thereby confounding the results of the neuroimaging analysis.

One might assume that the lower proportion of negative pictures viewed by PD patients was caused by increased harm avoidance, a key feature of depression [34, 35]. In fact, earlier studies have demonstrated that harm avoidance is increased in PD patients [29, 30]. Thus, the PD patients, who were associated with higher depression scores in the present study, might have tried to avoid viewing negative pictures due to increased harm avoidance, rather than decreased novelty seeking. However, this is not likely; if the increased harm avoidance associated with a depressive state contributes to a decreased frequency of viewing negative pictures, the depression score would have been identified as a mediating factor. However, our mediation analysis did not reveal a significant mediation effect, thereby ruling out this alternative interpretation.

Both the PD patients and HCs viewed more pictures under the uncertain condition than under the certain condition. This suggests that the motivation to resolve uncertainty was maintained in the PD patients. Although earlier studies of curiosity regarding trivial questions and uncertain images have commonly reported striatal activation [14, 36–38], Jepma et al. reported that the insula and anterior cingulate cortex, which are associated with conflict and arousal, were activated at the time of unclear stimulus presentation, during which curiosity was not yet satisfied [38]. They assumed that curiosity evoked by ambiguous stimuli is an aversive condition and induces an increase in arousal. Therefore, these cortical brain regions, which are relatively intact in PD patients, may be responsible for maintaining the motivational aspect to resolve uncertainty, even though the dopaminergic neurons and pathways have degenerated.

To the best of our knowledge, the present study’s findings are the first to demonstrate that intrinsic motivation is partly impaired in PD patients, possibly due to dopaminergic degeneration. A major limitation of the present study was that we lacked the opportunity to investigate medication-dependent performance changes. Although the present results revealed no correlation between levodopa equivalent dose and the proportion of pictures viewed in the experimental task (*r* = 0.26, *p* = 0.20), a direct comparison between on and off states using a within-subject design would be informative. Past studies have shown an inverted U-shaped relationship between dopamine-dependent cognitive abilities and dopamine levels [39, 40]. Thus, optimal levels of dopamine can remedy the general tendency of PD patients to avoid negative pictures, but both insufficient and excessive levels of dopamine can have adverse effects.

## Data Availability

The data are not publicly available due to informed consent restrictions concerning confidential patient information, but are available from the corresponding author on reasonable request.
